# Monitoring Urban Expansion and Loss of Agriculture on the North Coast of West Java Province, Indonesia, Using Google Earth Engine and Intensity Analysis

**DOI:** 10.1155/2022/3123788

**Published:** 2022-01-12

**Authors:** Laju Gandharum, Djoko Mulyo Hartono, Asep Karsidi, Mubariq Ahmad

**Affiliations:** ^1^School of Environmental Science, Universitas Indonesia (UI), Jl. Salemba Raya No. 4, Kampus UI Salemba, Jakarta Pusat, Jakarta 10430, Indonesia; ^2^Center of Regional Resources Development Technology (PTPSW), National Research and Innovation Agency (BRIN), Lantai 2, Gedung Geostech 820, Kawasan Puspiptek, Serpong, Tangerang Selatan, Banten 15314, Indonesia; ^3^Environmental Engineering Study Program, Faculty of Engineering, Universitas Indonesia (UI), Kampus Baru UI, Depok 16424, Indonesia; ^4^Department of Geography, Faculty Mathematics and Natural Sciences, Universitas Indonesia (UI), Kampus Baru UI, Depok 16424, Indonesia

## Abstract

Uncontrolled urban expansion resulting from urbanization has a disastrous impact on agricultural land. This situation is being experienced by the densely populated and fertile island Java in Indonesia. Remote sensing technologies have developed rapidly in recent years, including the creation of Google Earth Engine (GEE). Intensity analysis (IA) is increasingly being used to systematically and substantially analyze land-use/land-cover (LULC) change. As yet, however, no study of land conversion from agriculture to urban areas in Indonesia has adopted GEE and IA approaches simultaneously. Therefore, this study aims to monitor urban penetration to agricultural land in the north coastal region of West Java Province by applying both methods to two time intervals: 2003–2013 and 2013–2020. Landsat data and a robust random forest (RF) classifier available in GEE were chosen for producing LULC maps. Monitoring LULC change using GEE and IA has demonstrated reliable findings. The overall accuracy of Landsat image classification results for 2003, 2013, and 2020 were 88%, 87%, and 88%, respectively. IA outputs at interval levels for all categories showed that the annual change-of-area rate was higher during 2013–2020 than during 2003–2013. At the category level, IA results showed that the area of agricultural land experienced net losses in both periods, with net loss in 2013–2020 being 2.3 times greater than that in 2003–2013 (∼1,850 ha per year). In contrast, the built-up area made net gains in both periods, reaching almost twice as much in the second period as in the first (∼2,030 ha per year). The transition-level IA performed proved that agricultural land had been the primary target for the expansion of built-up areas. The most extensive spatial distribution of land conversion from agriculture to built-up area was concentrated in the regencies of Bekasi, Karawang, and Cirebon. These findings are intended to provide stakeholders with enrichment in terms of available literature and with valuable inputs useful for identifying better urban and regional planning policies in Indonesia and similar regions.

## 1. Introduction

On a global scale, it is predicted that urbanization will continue to degrade critical ecosystems far into the future [1], posing challenges for government, policymakers, and urban planners in reallocating resources [2]. The drastic effect of socioeconomic changes on the relationship between urbanization and agricultural land means that urban land expansion creates pressures on both national food security [3] and Sustainable Development Goal 2 (SDG 2) to achieve zero hunger, a target that will be challenging to achieve by 2030. This situation is being experienced by Indonesia, the world's fourth most populous country after China, India, and the United States.

Statistics Indonesia/Badan Pusat Statistik (BPS) records that Indonesia's population reached around 270 million in 2020, of whom more than 56% or approximately 151 million people live in Java Island [4]. The country's population is projected to reach nearly 280 million by 2045, with an urbanization rate of 72.9%. Most of these urban dwellers will live in urban areas of Java Island [5], even though the island of Java makes up only 7% of mainland Indonesia. Java Island also has a high economic growth of 5.55%, surpassing the national economic growth rate of 5.02% in 2019 [6]. Furthermore, Java Island contributed 50% of national agricultural production in 2015 [7].

According to Indonesia's Ministry of Agrarian Affairs and Spatial Planning, agricultural land in Indonesia, particularly rice fields, decreased by around 287,000 ha from 2013 to 2019, with 7.64 million ha remaining [8]. Java accounted for 90% of this decline [9]. Land conversion is typical, but it becomes challenging in the context of urban development and shrinking productive agricultural land where supply is limited and conversion is uncontrolled, resulting in negative consequences for climate and for human beings themselves. Efforts to anticipate and control this problem are critical. In this regard, a large body of literature has used remote sensing and geographic information systems (GIS) in its research [10, 11]. Remote sensing and GIS have advantages such as they are able to cover wide areas in timely, accurate, and affordable ways that allows to replacing mapping that relies on field surveys alone [12].

Access to the Google Earth Engine (GEE) platform since 2010 has made working on remote sensing applications more attractive [13]. This online-based environmental monitoring platform combines a multi-petabyte catalog of satellite imagery, such as Landsat and Sentinel, with global-scale analysis capabilities. These resources are available to scientists, researchers, and developers to enable them to detect and measure changes on Earth's surface [13]. GEE provides various machine-learning algorithms that can be used to classify satellite imagery data [14], one of the most common and extensively used of which is the random forest algorithm (RF) [15, 16]. The advantage of using RF is its level of high accuracy, with an average accuracy rate of more than 90% [15, 17]. Landsat is satellite image data widely used in various LULC change studies [15, 18–21]. The benefits of using Landsat data are that it is available for a long time span, stable, and free [11]. It provides appropriate spectral and spatial resolution, has a time revisit of 16 days, and is not as complex as synthetic-aperture radar (SAR) imagery [12].

Many previous studies focus only on characterizing LULC change without systematically analyzing it [22]. Usually, the cross-tabulation method has been proposed in those studies to indicate changes in land use relative to the size of the land-use categories so that the main signals of frequent transitions in land use between points in time can be identified [22]. Aldwaik and Pontius [23] improved on the cross-tabulation approach by introducing the intensity analysis (IA) technique. This quantitative technique is used for determining the size and intensity of transitions in different land categories over two or more time intervals by computing deviations between observable changes and hypothetical uniform changes [24]. IA features a top-down hierarchy at the interval, category, and transition levels and a layered progressive system that demonstrates the process of LULC change. At the same time, IA evaluates the process of LULC change by providing evidence of stability. Stable LULC conversion may not necessarily lead to sustainable development, but it could provide an essential basis for it [25].

There have been many published articles on the conversion of agricultural land to nonagricultural use in Indonesia, especially in Java, but generally these cover only one administrative area, usually at the regency level [26–28]. Meanwhile, research that covers a larger area is scarce but includes one study that focuses solely on statistical evidence to describe the issue of agricultural land conversion and its consequences for regional spatial planning in West Java's north coast [29]. Furthermore, to the best of the authors' knowledge, there has been no study of the conversion of agricultural land to urban areas using GEE and IA simultaneously and applied to Java and Indonesia.

To fill this research gap, this study aims to monitor and understand the dynamics of the conversion of agricultural land to urban areas in Java systematically and in depth using GEE and IA. To represent Java, the north coast of West Java Province was chosen as the study area. IA was conducted across two time intervals: 2003–2013 and 2013–2020. During the period 2003–2013, Indonesia experienced a political power transition from the “New Order” era that had ended in 1998 to the “Reformation” era. Meanwhile, the period of 2013–2020 was the Reformation era in which power shifted to President Joko Widodo (known as Jokowi). Jokowi's most significant policy concerns the building of infrastructure throughout the country to deliver equitable development, to improve people's lives, and to attract investors. Findings are expected to contribute to the enriching of regional and urban planning studies in Indonesia and similar areas.

The remaining paper is organized as follows.  Section 2 describes the study area. The use of Landsat imagery and RF on GEE and land change analysis using IA are explained in Section 3. LULC maps as image classification results and their validation values are presented in Section 4. This section also presents LULC change in cross-tabulation matrices and IA analyses.  Section 5 discusses the effectiveness and the limitation of using GEE, the distribution pattern of LULC, the remaining agricultural land, food estate, and Indonesia's global commitment to improving world climate. Finally, the conclusion and future work are presented in Section 6.

## 2. Study Area

**Figure 1:**
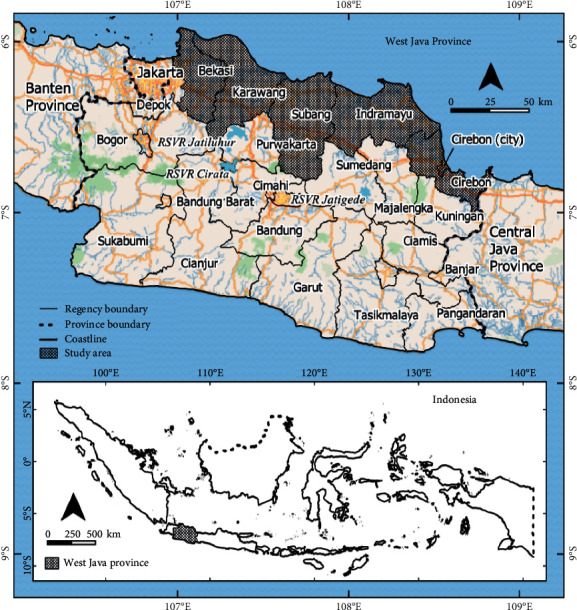
Study area.

The north coast of West Java Province (stretching from 106° 58′ 6.88″E, 7° 0′ 22.69″ S to 108° 50′ 48.77″E, 5° 55′ 5.33″ S) was selected as the study area because it is one of the nation's most important food production areas. Despite this critical contribution, the area is experiencing rapid land conversion from agricultural to nonagricultural land [29]. The study area is ±8,565.2 km^2^ and administratively consists of the regencies of Bekasi, Karawang, Subang, Indramayu, and Cirebon and the municipality of Cirebon (see Table 1 and Figure 1). In this study, because Cirebon Municipality is relatively small (0.5% of the study area) and was historically part of Cirebon Regency, the two regions are merged in the calculations.

In 2019, West Java Province was inhabited by 49.32 million people, making it Indonesia's most populous province. Nearly 12 million of its residents (24.24 percent) live on West Java's north coast, with an estimated annual growth rate of 1.39 percent from 2010 to 2019 and a population density of 2,827 people per square kilometer [30].

West Java Province is the country's third largest rice producer, with a contribution of 9.08 million tonnes (16.63%) [31]. Almost half of this amount is produced in the study area [32]. In this area, rice is generally grown in irrigated fields, covering an area of approximately 444,233 ha or 51.9 percent of the total. These rice fields are irrigated by water sourced from two of the nation's largest reservoirs, the Jatiluhur and the Jatigede (Figure 1).

The altitude of the study area ranges from 0 to 2,196 meters above mean sea level (AMSL), with 65.53 percent at 0–25 meters AMSL and slopes of 0–8 percent extending along the coast in the northern part of West Java. Just 1% of the mountainous part of the region has an elevation of more than 1,000 meters AMSL with steep slopes (>45%). The land cover in this higher region is forest and is located in the southern part of the Karawang and Subang regencies. The study area is characterized by fertile alluvial lowlands as agricultural purposes, with annual rainfall ranging from 1,000 to 2,500 mm per year [33].

## 3. Materials and Methods

### 3.1. Materials

Optical Landsat imagery from the United States Geological Survey (USGS) was the primary data source for this study. The appearance of cloud cover in the images is the biggest challenge when using Landsat data. Clouds are nearly always present throughout the year across large areas, archipelagos, and tropical climates like Indonesia. Therefore, to get cloud-free images, pixels were selected from long-term archival Landsat data. The Landsat data circa 2003 was sourced from the Landsat 7 Enhanced Thematic Mapper Plus (ETM+) satellite, while data for 2013 and 2020 were obtained from the Landsat 8 Operational Land Imager (OLI) satellite. A total of 231 scenes from Landsat images were processed for this study.

In addition, reference data used in this study were in the form of LULC maps, very high spatial resolution satellite images, and field survey data. LULC maps for 2003, 2013, and 2020 were sourced from the Ministry of Forestry and Environment of the Republic of Indonesia (MoEF) accessed through https://dbgis.menlhk.go.id. The very high spatial resolution satellite imagery for 2003, 2013, and 2020 was available from Google Earth Pro software. These data have a spatial resolution of 15–30 cm [34]. Finally, reference data were drawn from a field survey carried out in June 2019. From these three sets of reference data, some data were used as training samples in the Landsat image classification process, while the rest were used for validation.

As ancillary data, this study used digital topographic vector map data (1 : 25,000) from Indonesia's Geospatial Information Agency (https://tanahair.indonesia.go.id/portal-web), administrative boundaries vector maps (1 : 25,000) and statistical data from BPS, and digital elevation Shuttle Radar Topography Mission data from the USGS.

### 3.2. Methods

**Figure 2:**
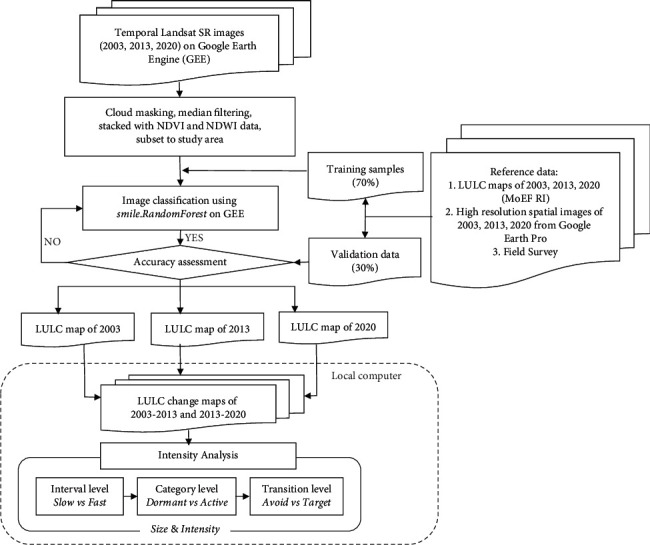
Workflow for analyzing land change.

The workflow used in this study is depicted in Figure 2.

#### 3.2.1. Image Classification on Google Earth Engine (GEE) and Accuracy Assessment

The GEE Landsat images used were surface reflectance (SR) products. For circa 2003, the collections of Landsat SR imagery were selected from Landsat 7 ETM+, while the 2013 and 2020 images came from Landsat 8 OLI. Reserving cloud-free pixels of the image was done by running the clouds masking function. Only visible (VIS), near-infrared (NIR), and short-wave infrared (SWIR) bands were used in this study, all of which had spatial resolution of 30 m. A median filter was then applied to obtain representative pixels for each location of each spectral band from each year. To achieve higher accuracy in image classification results, each of the 2003, 2013, and 2020 bundles of Landsat data was stacked with the normalized difference vegetation index (NDVI) and the normalized difference water index (NDWI) data for the corresponding years and then clipped to the study area. These data on GEE were the Landsat 7 32-Day NDVI Composite and Landsat 8 32-Day NDWI Composite data.

The reference data were then prepared, with 70% being used as training samples to classify the Landsat images for 2003, 2013, and 2020 and the remainder being used to validate the image classification results. The reference data were distributed at random points generated from the MoEF's LULC maps and some taken from the field survey data. These points represented the five LULC categories addressed in this study: (1) agriculture (rice fields, fishponds, and plantation), (2) forest, (3) built-up (settlements, industrial areas, roads, and other artificial land-use/land-cover), (4) shrubs/bushes, and (5) water bodies (rivers, lakes, canals, and others). Points were distributed across the study area, with a total number of 10,000 points taken from each data year. Prior to use, the data were visually checked using historical high-resolution images from Google Earth Pro.

Based on the training samples, image classification was applied to Landsat images to produce LULC maps for 2003, 2013, and 2020 using the RF algorithm. Subsequently, each RF classification result was subjected to postprocessing, which included weighted refining with a 3 × 3 moving window to eliminate “noise” and editing of misclassified parts to create final LULC maps.

Accuracy assessment was then conducted for each of the LULC maps separately. As previously mentioned, the validation data used in this process were 30% of the reference data, selected randomly. An error matrix was employed in accuracy assessment to produce overall accuracy values for each LULC map for 2003, 2013, and 2020.

#### 3.2.2. Land Change Analysis

In vector format, the final LULC and administrative boundary maps were superimposed onto each other. This process aimed to determine the spatial distribution and quantity of land conversion areas for 2003–2013 and 2013–2020 either for the entire study area or for each regency. The quantification results in the form of transition matrices/cross-tabulation matrices became the input for IA for the time intervals 2003–2013 and 2013–2020. IA was used to examine LULC change by combining three levels of analysis (interval, category, and transition) into one unified framework. These characterized the pattern of change appropriately at a more detailed level (discovering processes and causes of change) over several different time intervals, for application as references in land-use planning [23, 24]. In this study, IA was examined at the levels of the entire study area and each of its regencies.

For the first level, the interval level measures the total scale and speed of change of all LULC categories in different time intervals. Equation (1) measures how the size and rate of change varies annually [*S*_*t*_,] during each time interval [*Y*_*t*_, *Y*_*t*+1_]. In equation (1), *t* denotes the index for a time point and *Y*_*t*_ for the year at that time point. The changes in each time interval are then compared with the uniform intensity *U* (equation (2)) for the whole temporal extent. Annual change of a time interval is considered slow when it is smaller than the uniform change, while a fast annual change is identified when the annual change is larger than the uniform change.

(1) $$\begin{array}{c} S_{t}=\frac{\left(\text{size of change during }\left[Y_{t},Y_{t+1}\right]\right)}{\left(\text{size of spatial extent}\right)\left(\text{duration of }\left[Y_{t},Y_{t+1}\right]\right)}100\text{\%,} \end{array}$$

(2) $$\begin{array}{c} U=\frac{\left(\text{size of change during all intervals}\right)}{\left(\text{size of spatial extent}\right)\left(\text{duration of all intervals}\right)}100\text{\%.} \end{array}$$

The second IA level is the category level. It produces the change trends for each individual LULC category. This makes comparisons among the types of LULC transition in terms of the intensity of change, where the loss intensity *L*_*ti*_ (equation (3)) from category *i* and the gain intensity *G*_*tj*_ (equation (4)) to category *j* compares to a uniform intensity *S*_*t*_ during each time interval [*Y*_*t*_, *Y*_*t*+1_]. An active annual change in an LULC category is identified as occurring if it is larger than the uniform intensity of change in a time interval, while dormancy is identified when the annual change of an LULC category is smaller than the uniform intensity of change in a time interval [35].

(3) $$\begin{array}{c} L_{ti}=\frac{\left(\text{size of loss of }i\text{ during }\left[Y_{t},Y_{t+1}\right]\right)}{\left(\text{size of }i\text{ at time }Y_{t\;}\right)\left(\text{duration of }\left[Y_{t},Y_{t+1}\right]\right)}100\text{\%,} \end{array}$$

(4) $$\begin{array}{c} G_{tj}=\frac{\left(\text{size of gain of }j\text{ during }\left[Y_{t},Y_{t+1}\right]\right)}{\left(\text{size of }j\text{ at time }Y_{t+1}\right)\left(\text{duration of }\left[Y_{t},Y_{t+1}\right]\right)}100\text{\%.} \end{array}$$

The last level of IA, the transition level, quantifies the size and intensity that a category gains from other categories during a certain time interval. It compares transition intensity *R*_*tij*_ during an analyzed time interval (equation (5)) from category *i* to category *j* to a uniform transition intensity *W*_*tj*_ (equation (6)), given the gain in category *j*. The transition level answers the issue of which transitions in a given time interval are notably intensive, which is useful for understanding the transition from a certain category, *i*, to another category, *j*. In this study, the transition level focuses on built-up and agricultural areas.

(5) $$\begin{array}{c} R_{tij}=\frac{\left(\text{size of transition from }i\text{ to }j\text{ during}\left[Y_{t},Y_{t+1}\right]\right)}{\left(\text{size of }i\text{ at time }Y_{t}\right)\left(\text{duration of }\left[Y_{t},Y_{t+1}\right]\right)}100\text{\%,} \end{array}$$

(6) $$\begin{array}{c} W_{tj}=\frac{\left(\text{size of gain of }j\text{ during}\left[Y_{t},Y_{t+1}\right]\right)}{\left(\text{size of not }j\text{ at time }Y_{t+1}\right)\left(\text{duration of}\left[Y_{t},Y_{t+1}\right]\right)}100\text{\%.} \end{array}$$

In this study, IA was carried out using a free computer program developed by Aldwaik and Pontius Jr. It was downloaded from https://sites.google.com/site/intensityanalysis and run on Microsoft Excel.

## 4. Results

### 4.1. LULC Maps, Patterns, and Validation Results

**Figure 3:**
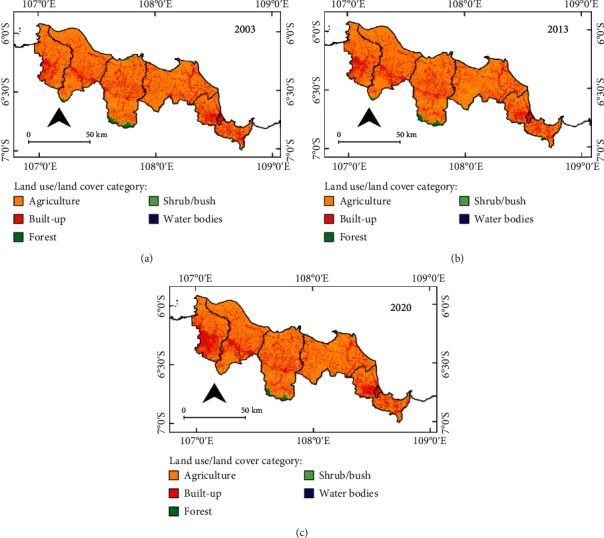
LULC maps for (a) 2003, (b) 2013, and (c) 2020 derived from Landsat imagery.

**Figure 4:**
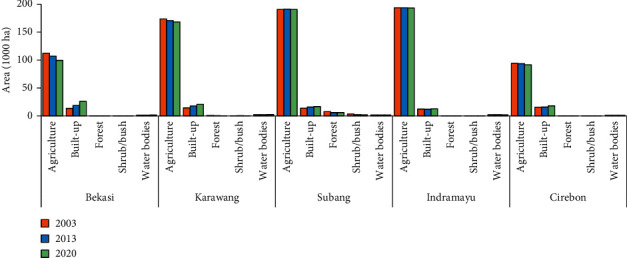
Area of LULC in 2003, 2013, and 2020 for each category at regency level.

Figure 3 features maps of the study area showing distribution of the five LULC categories agriculture, built-up, forest, shrub/bush, and water bodies for the years 2003, 2013, and 2020. The maps were created from the Landsat image classifications using the RF algorithm provided on GEE. The accuracy assessment results for these three maps show an overall accuracy of 88%, 87%, and 88% for 2003, 2013, and 2020, respectively. The results of the calculations of LULC area for each category in each of the three years are presented in Table 2.  Figure 4 presents bar graphs for the area of each LULC category in each regency/municipality.

Figure 3, Table 2, and Figure 4 show that LULC is dominated by the agricultural category (more than 744,000 ha or 86%) and that this category is evenly distributed across the study areas for the three years. In 2020, the largest area of agricultural LULC is in Indramayu Regency (193,514 ha), followed by Subang Regency (190,847 ha), while the smallest is in Cirebon Regency/Municipality (91,666 ha). Built-up area was the second-largest LULC category, with an average area of 9.5%. This built-up LULC is scattered throughout the study area, but its development is concentrated in the southwest of Bekasi Regency, from west to east in the central area of Karawang Regency, and in Cirebon Municipality and its surrounding areas. Bekasi Regency had the highest proportion of built-up area to total area (20%) in 2020, while Indramayu Regency had the lowest (5.2 percent). Other categories such as forest, shrub/bush, and water bodies across the region each occupied 1% more or less of the study area on average. Forest, in particular, is found only in the southernmost part of Subang Regency, a hilly region with steep slopes.

### 4.2. LULC Change Dynamics

Based on the superimposition of the three LULC maps presented in Figure 3, transition matrices were generated for the two time intervals 2003–2013 and 2013–2020 for the entire study area (as shown in Table 3) and its regencies. The matrices show each LULC category where there was persistent and change in areas in two different years. The matrices also summarize the total area of gross gain and loss for each category of LULC. Of the 2003–2013 matrix, the total agriculture area shows 764,574.22 hectares in 2003 (see the third row in Table 3) and it remains persistent at 732,339 hectares in 2013. The difference in the agriculture area between 2003 and 2013, 32,235.22 hectares, is described as a gross loss of agriculture. A gross loss of agriculture in 2013 was made up of forest (1,068.38 ha), built-up (27,439.80 ha), shrub/bush (2,548.14 ha), and water bodies (1,178.90 ha). The third column of Table 3 depicts that the total area of agriculture in 2013 is 756,698.39 ha, of which 732,339 ha remains persistent, while 24,359.38 ha is its gross gain consisting of 3,465.76 ha, 16,624.67 ha, 3,294.88 ha, and 974.07 ha for forest, built-up, shrub/bush, and water bodies, respectively. Of those values, agriculture gross loss has a bigger value than gross gain, where the difference between the two is ∼7,876 ha, so it can be said that agriculture during the 2003–2013 period (within ten years) experienced a net loss of ∼7,876 ha or an average of ∼790 ha per year. On the other hand, a net gain will be obtained if the gross gain value of a certain category is bigger than its gross loss value.

### 4.3. Intensity Analysis (IA)

#### 4.3.1. Interval Level

**Figure 5:**
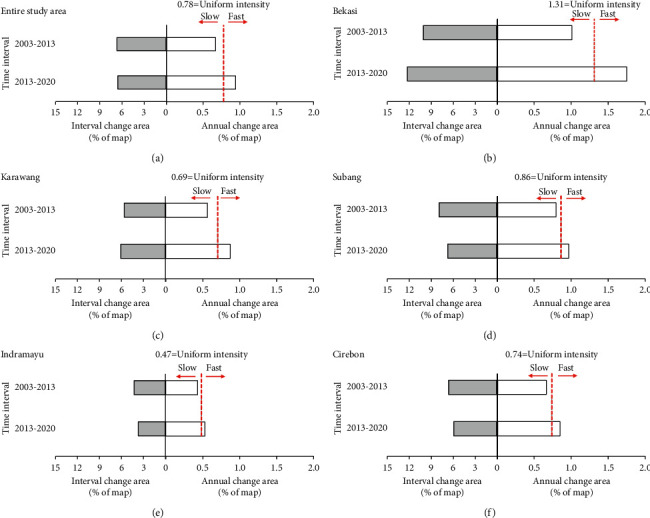
Interval levels of IA for the two periods 2003–2013 and 2013–2020 in (a) the entire study area and (b–f) at regency level.

**Figure 6:**
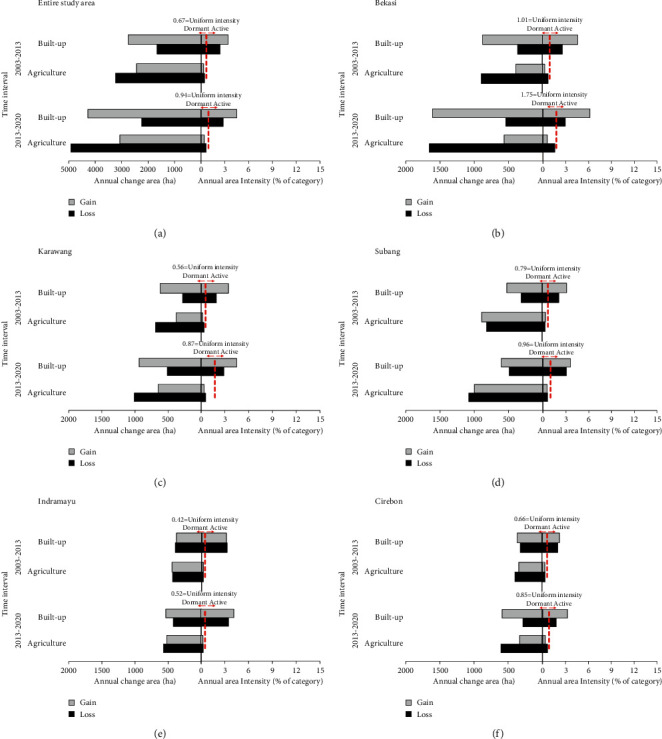
Category-level IA for time intervals 2003–2013 and 2013–2020 for the entire study area (a) and for each regency (b–f).

The interval levels of the IA results for the two periods, 2003–2013 and 2013–2020, are depicted in Figure 5. Furthermore, this figure presents interval level charts for the entire study area (Figure 5(a)) and for each regency/municipality, i.e., Bekasi, Karawang, Subang, Indramayu, and Cirebon (Figures 5(b)–5(f)). The red vertical dashed line in Figure 6 represents the uniform intensity value, which is the mean change rate of all LULC categories in the two time intervals calculated using equation (2).

Figure 5(a) shows that for the entire study area, the percentage of interval change area rates of all categories (bars on the left of the central axis) for the two time intervals are almost the same (6.66% compared with 6.56%, for 2003–2013 and 2013–2020, respectively). However, the annual change area rates of all categories (on the right of the central axis) reveals that the rate in the second time interval is greater than in the first (by 0.67 percent), despite the fact that the second time interval is just seven years long and is therefore shorter than the first period. Thus, LULC change in the second period can be said to be faster than in the first period. This is also indicated by the rate of change in the second period exceeding the uniform intensity value of 0.78%.

In considering the interval levels at the regency scale (Figures 5(b)–5(f)), it can be seen that in Bekasi and Karawang regencies, the area change intervals were smaller in 2003–2013 than in 2013–2020, at 10.07% versus 12.24% and 5.60% versus 6.08%, respectively. Meanwhile, the regencies of Subang, Indramayu, and Cirebon show a different pattern where the area change interval in the 2003–2013 period is greater than that in the 2013–2020 period. The values of the area change intervals for Subang, Indramayu, and Cirebon regencies in the first and second periods are 7.92–6.75%, 4.25–3.67%, and 6.63–5.94%, respectively. Although the area change intervals are different across the regencies, the annual change area rates for all regencies in the second period are greater than that in the first period. Hence, it can be interpreted that across all regencies, the LULC change in the second period occurred faster than that in the first period, as indicated by the values of the annual change area rates in that period exceeding the corresponding uniform intensity values.

#### 4.3.2. Category Level

The area of annual gain and loss of each category during the two periods for the entire study area as IA results at the category level are revealed in Figure 6(a), while the equivalent data for each regency are shown in Figures 6(b)–6(f). Since our research purpose was to understand the impact of built-up area change on agricultural land, and because the other categories show small amounts of LULC change, the categories of forest, shrub/bush, and water bodies are not presented. Based on Figure 6(a), the annual change (bars on the left) shows that the agriculture category experienced a net loss in all time intervals, with the net loss for 2013–2020 (∼1,850 ha) being 2.3 times greater than for 2003–2013 (∼790 ha). Meanwhile, the built-up category shows the opposite trend, in that both periods experienced a net gain. The net gain in the second phase (∼2,030 ha per year) was 1.9 times greater than that in the first (∼1,080 ha per year). The bars on the right indicate the annual area intensity of agriculture and built-up in two periods. The red dashed line represents the uniform change intensity derived using equation (4) for each time interval. The change is active when a bar extends beyond the uniform intensity line. The change is dormant if a bar falls before the uniform intensity line. The gains of built-up were active during all time intervals, while agriculture's gains were dormant. Furthermore, the built-up category had more active gains for the second period rather than the first. Meanwhile, the agriculture category experienced more dormancy in the second period than in the first.

At the regency level (Figures 6(b)–6(f)), gains and losses for built-up and agriculture categories for both time intervals appear to have a similar pattern to the entire study area, in which the annual area change in 2013–2020 was greater than that in 2003–2013. In all periods, the value of the annual area change intensity for the built-up category exceeds the uniform intensity value so can be said to be active. In contrast, agriculture is dormant because the value of the annual area change intensity is smaller than the uniform intensity. However, the value of net gains and net losses of the annual change in area for these two categories at the regency level varies. In Bekasi Regency, net gain in built-up area was greater than that in the other regencies in the two periods, i.e., 510 ha for 2003–2013 and 1,067 ha for 2013–2020. Meanwhile, a net loss for agriculture of 505 ha in 2003–2013 and 1,091 ha in 2013–2020 was identified.

Similar patterns but with lower values occurred in Karawang and Cirebon regencies. In Karawang, net gain in built-up area was 337 ha and 423 ha in the first and second periods, respectively, while net loss in agriculture was 310 ha and 358 ha, respectively. In Cirebon Regency in the first and second periods, respectively, net gain in built-up area was 43 ha and 304 ha, while net loss of agriculture was 55 ha and 273 ha.

A slightly different pattern is revealed in Subang and Indramayu districts in the first period. In Subang in that period, the agriculture category experienced a net gain of 73 ha, while built-up area experienced a net gain of 210 ha. Meanwhile, agriculture in Indramayu experienced a net gain of 10 ha and built-up experienced a net loss of 18 ha. However, in the second period, these two regencies had the same pattern as the other regencies. For Subang and Indramayu regencies, agriculture experienced a net loss of 81 ha and 273 ha, respectively, while the built-up category experienced a net gain of 117 ha and 304 ha.

#### 4.3.3. Transition Level

**Figure 7:**
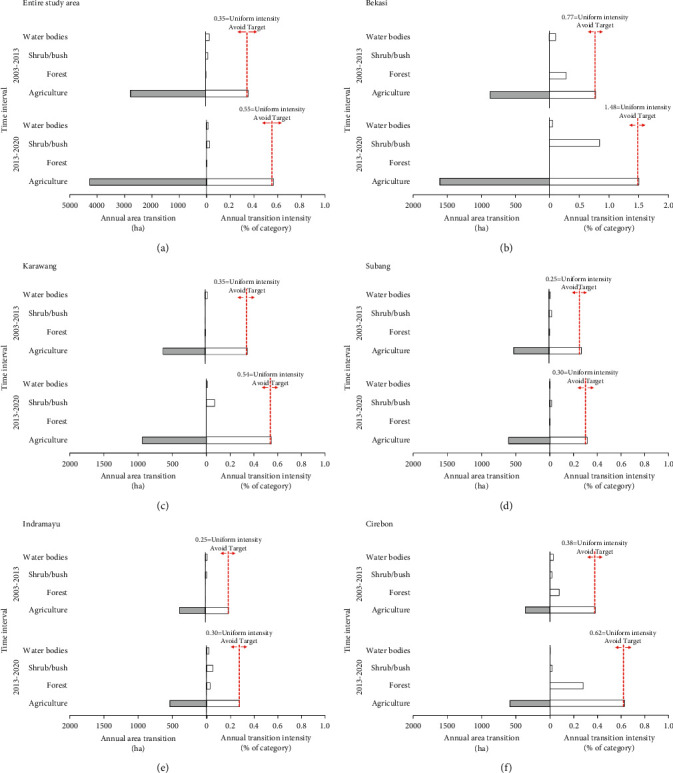
Transition-level IA for the built-up category for the entire study area (a) and per regency (b–f) for the two time periods 2003–2013 and 2013–2020.

**Figure 8:**
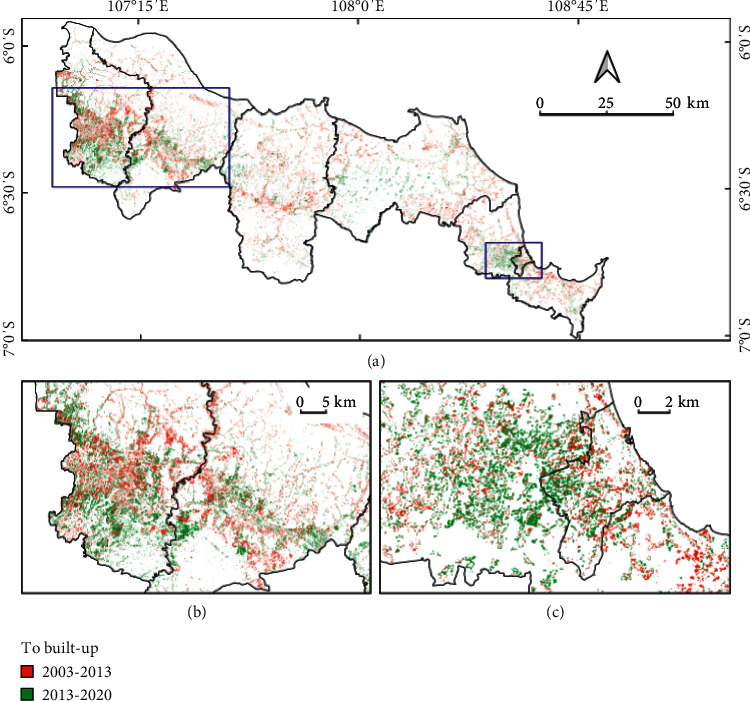
Spatial distribution of LULC transition to built-up areas in the entire study area (a) and to places undergoing significant changes in Bekasi and Karawang regencies (b) and Cirebon Regency and Municipality (c).

Figure 7 illustrates the transition level of IA, with Figure 7(a) covering the entire study area and Figures 7(b)–7(f) presenting results at the regency level. These six graphs show the analysis of gain to the built-up category from the other categories for the two time periods. These graphs show that the agriculture category contributed a much larger quantity of land to the gain in built-up area than the shrub/bush, forest, and water bodies categories. Although the second study period was shorter, the contribution of agriculture to gain in built-up land within it is greater than in the first period. The annual difference in area transition from agriculture to built-up land across all study areas for the two time intervals was ∼1,500 ha (Figure 7(a)). The largest contribution to this difference came from Bekasi Regency (739 ha), followed by Karawang (320 ha), Cirebon (227 ha), Indramayu (158 ha), and Subang (88 ha). Also reflected by the annual transition intensity values is that, for both time periods, the uniform intensity value was exceeded. This implies that the agriculture category was the target of the expansion of the built-up category. Meanwhile, other categories can be seen to have been avoided, given that the intensity of their annual transition was lower than the uniform intensity. Spatial LULC change to the built-up category is shown in Figure 8.

## 5. Discussion

Google Earth Engine (GEE) has been proven to be a sophisticated platform that is easy for both experts and nonexperts to use via Internet browsers and with JavaScript and Python programming languages. In this study, the availability of sample scripts on GEE and other online sources, as well as excellent explanations, shortened the time required to develop appropriate scripts. With the parallel processing capabilities of Google's cloud, the processing of 231 Landsat scenes in this study was accomplished in minutes. However, GEE has its limits. Time, memory, and storage limits are the most common issues encountered while running a script on the GEE environment [36]. Moreover, Navarro [36] also reported these limitation as follows: Unfortunately, on-demand (interactive) tasks cannot run last more than five minutes. Instead, batch tasks can be used. And memory problem occurs when running some commands on huge data images, while storage limit problem appears when saving the image output on Google Drive or Google Cloud with a free Google account that limits to 15 gigabytes. Furthermore, in this study, not all of the processes were completed entirely using GEE. Some of the work, including the IA, was performed on a local computer. Overall, research activities were designed to be efficient and cost effective.

The results of the IA at interval levels for the entire study area (the north coast of West Java) show that the annual area change rate in the second period (2013–2020) was greater than that in the first period (2003–2013) and in both periods exceeded uniform intensity. Bekasi Regency was the largest contributor at the regency level. The annual area change rate for Bekasi Regency in 2003–2013 and 2013–2020 was significantly larger than the rate for the entire study area, i.e., 10.07% and 12.24%, respectively, compared with 6.6% and 6.56%. Bekasi, along with Tangerang, Depok, and Bogor, is one of the peri-urban areas of the capital city of Jakarta. The entire megapolitan area is known as the Jakarta metropolitan area or Jabodetabek. Jakarta has greatly influenced its peri-urban area, leading to rapid development over the past three decades compared with other regions [37]. In Bekasi Regency, this can be illustrated by, among other indicators, population, gross regional domestic product (GDRP) per capita, and the Human Development Index (HDI). The population growth and growth rate of Bekasi Regency in 2020 were 3.11 million and 1.19%, respectively [38]; its GRDP per capita (at constant prices) for 2010–2020 was $4,205, surpassing other districts and the whole of West Java Province ($1,822) [38,39]. Meanwhile, its average HDI in 2016–2020 ranks second after Cirebon City, with a value of 74.37 [38].

Based on the results of IA at the category level, it can be seen that agriculture has experienced a huge net loss in both study intervals. Net loss in the second period was 2.3 times greater than that in the first period, at ∼1,850 ha per year. Meanwhile, the built-up category experienced a large net gain in both periods, with the second period's gain being almost twice that of the first period (∼1,080 ha per year). These findings align with those of IA at the transition level, which reveals that the agriculture LULC category has been the primary target for urbanization. Agricultural land in the study area was the target of land conversion because it has a lower land rent value than the land use category for services/commercial, industrial, and housing [40]. In addition, the relatively flat region and the availability of adequate infrastructure and human resources make it an allure for the expansion of housing, offices, and industries in the study area. Furthermore, it is much more expensive to convert water bodies and the forests are not touched because their location is irrelevant to the purpose of conversion.

The Cikopo–Palimanan Toll Road (also known as the Cipali Toll Road) is one of the contributors to this LULC transformation. The 116 kilometer toll road, which is part of the Trans-Java toll road project, has operated since 2015 and connects the regencies of Purwakarta, Subang, Indramayu, and Cirebon [41]. Exit toll points along the road stimulate new economic growth [42, 43], accelerating the shift from farmland to developed areas. Meanwhile, Bekasi and Karawang were influenced much earlier by the existence of toll roads including the Jakarta–Cikampek (Japek) Toll Road, which was built in 1988 connecting Jakarta, Bekasi, Karawang, and Purwakarta.

In terms of quantity, the largest land conversion from agricultural to built-up areas occurred in Bekasi Regency compared with other regencies. The factor that most determines LULC is population density because of its implications for the expansion of residential areas. If this is not controlled, Bekasi Regency is predicted to experience a rice deficit in 2025 [44]. In addition, the settlement area deviated by 37% from Bekasi's spatial planning map. Other regencies had comparable changes in land function from agriculture to built-up land, but with differing magnitudes, with the degree of the transition being larger in 2013–2020 than in 2003–2013.

Interestingly, the dynamics of LULC change to the built-up category in the study area was also concentrated in the border area between Cirebon Municipality and Cirebon Regency (Figure 8(c)), even though these regions are located about 258 km east of Jakarta and 130 km northeast of the capital city of the province, Bandung. This can be understood as reflecting Cirebon's status as regional capital during the Dutch colonial era until 1959, after which the urban area became a new municipality and was designated as a regional activity center serving its surrounding area [45]. Furthermore, Cirebon is transected by the Java Northern Coastline (Jalur Pantura), which is one of the essential national transport corridors in Java Island. The existence of the Cipali Toll Road has also accelerated development in the area [43]. Given these conditions, new urban growth centers have developed both on the outskirts of the municipality and in its surroundings, such as in Karangsembung as the gateway to and from Jakarta-Bandung, Kedawung as a residential area, and Weru for industrialization activities [45].

Moreover, with Subang Regency and Majalengka Regency, Cirebon will be assigned to a “new metropolitan” area known as the Rebana Triangle [46], and it is being proposed by the local government to central government to become a special economic zone (Kawasan Ekonomi Khusus). This area is supported by infrastructure that has been, is being, and will be built, such as airports, seaports, and toll roads. Kertajati is an international airport in Majalengka Regency, which has been operating since 2018. Work on the Patimban seaport in Subang Regency was inaugurated in 2020 and is expected to be fully completed by 2027. The project is valued at Rp43.2 trillion and will span 369 hectares and a reserve area of 356 hectares with a total cumulative container capacity of 7.5 million twenty-foot equivalent units (TEUs) [47].

What is described above suggests that economic activities that have an impact on land requirements for infrastructure, industry, and settlements in the study location were more dynamic in the second period than that in the first period. Nationally, this was in line with the policy of the central government at those times. Infrastructure budget during the presidency of Susilo Bambang Yudhoyono (SBY) was Rp99 trillion in 2010 and Rp178 trillion in 2014 [48]. This figure rose sharply in the era of President Joko Widodo (Jokowi), and at the beginning of his administration in 2015, the infrastructure budget allocation reached Rp290 trillion, an increase of 63 percent compared with 2014. The budget continues to increase every year and was recorded as Rp415 trillion in 2019 [49].

One of Java's most significant infrastructure projects is constructing the Trans Java toll road network, which connects the western and eastern part of the island [41]. The new portion of Trans Java Toll Road runs for 1,350 kilometers, linking the western part of West Java Province to the eastern part of East Java Province, and is planned to be extended to Bali [50]. Pantura is where all the prime trading and industrial infrastructure located, so it is most profitable for private sectors to develop their industrial complex and facilities along the northern corridor. The study area has good proximity to Tanjung Priok, the main export harbor and right on the Indonesia East-West land transport corridor. Hence, it becomes the most economical location for industrial facilities.

Based on the above facts, it is likely, particularly in Java, that agricultural land will continue to experience conversion to built-up areas, whether planned or not, unless the government changes the main drivers for the land conversion, i.e., the cost advantage of industrial infrastructure. It appears that the efforts to control this conversion, as regulated in Law Number 41 of 2009 concerning Protection of Sustainable Food Agricultural Land and related regulations, have not been effectively implemented. A variety of factors are involved in this problem: (1) not all provinces and regencies/municipalities have completed/updated their regional regulations, in this case, the regional spatial planning regulation under which the location of sustainable agricultural land must be determined; (2) regional developments, specifically basic infrastructure, require a large amount of land resources; (3) LULC change has taken place independently and without the stipulated permits [51]; and (4) there is limited availability of appropriate spatial information.

The government's national strategic projects (NSPs) and industrial facilities outside Java, in addition to equitable development throughout the country, are also aimed at reducing pressure on land conversion on the island of Java. A new government NSP, known as food estate (FE), is also developed to improve the food security of an area so that there is equity and strengthen national food security and is also expected to become a new food barn outside Java [52]. In the years 2020–2024, this food estate program is being implemented in Kalimantan Island, Sumatra Island, and East Nusa Tenggara Province [53]. In Central Kalimantan Province, the food estate covers roughly 30,160 hectares and will be increased to 168,000 hectares in areas of both current and new rice fields. Rice production in the food estate pilot project area (2,000 ha) varies between 3 and 4 tonnes per hectare [53], which is still lower than in Java, where it is roughly 4.7–5.7 tonnes per hectare [54]. The food estate in Kalimantan was developed on the site of a former mega-rice project (MRP) set up in 1996 on land that was formerly a peatland [52]. This project was deemed a failure, and it left a slew of problems including environmental concerns and contributed to the world's worst land fires in 1997/1998 [55]. In 1997, these huge fires burned 4,740 km^2^ in the MRP area, releasing 0.19–0.23 gigatonnes of carbon into the atmosphere [56].

Scholars have expressed concerns about claims that the large-scale food estate program will supposedly not suffer the same failures as the MRP project. They suggest that it might harm Indonesia's efforts to meet its global commitment to improving world climate through its nationally determined contribution (NDC) by reducing deforestation and improving peatland management [57]. NDCs represent each country's efforts to decrease national emissions and adapt to climate change consequences. As is well known, Indonesia's NDC adopted the Paris Agreement in 2016, pledging to decrease its carbon emissions by 29–41 percent by 2030 compared with business as usual, with the higher end of this range to be attained with international collaboration [58]. Indonesia has also committed that it would achieve net zero emissions (NZEs) or carbon neutral, establishing a balance between greenhouse gas emissions generated and emissions removed from the atmosphere, by the year 2070 [59].

At the present time, it remains vital to preserve the existence of agricultural land in Java. In addition, in terms of maintaining soil fertility and increasing agricultural productivity, sustainable agricultural practices need to be improved. However, land fertility and high productivity are meaningless if there is no agricultural land remaining; the conversion of agricultural land to nonagricultural therefore needs to be more strictly controlled. The availability of spatial data and the use of appropriate analysis methods are needed to monitor agricultural land periodically and precisely. Landsat data, GEE, and IA are cost-effective packages that can fulfill this requirement. Using such tools, regional development can operate in line with the goals of sustainable development, where the benefits obtained not only address current needs but can also ensure that all resources can sufficiently meet the needs, including for food, of future generations.

## 6. Conclusion

Using GEE and IA has delivered reliable results in monitoring LULC change relating to urban development and shrinking agricultural land on the north coast of West Java Province. Although GEE performed well, it also has limitations, especially in time, memory, and standard output storage capacity. Furthermore, not all of the work was done entirely on GEE; other sections, such as IA analysis, were completed on a desktop computer. This study is focusing on determining the pattern of change rather than the causes or drivers of change. IA results show that, even though the time interval is shorter, LULC change dynamics are even greater in 2013–2020 than in 2003–2013. The categories of agricultural land and built-up areas dominate the dynamics of LULC change, with agricultural land being the target of expansion in built-up areas. The net gain in built-up area is almost twice as large in the second period as in the first (∼2,030 ha per year), while the net loss of agricultural land in the same period is twice as large as in the first period (∼1,850 ha per year). This situation is related to the current focus of the Indonesian government on infrastructure development.

The nature of the conversion of agricultural land to built-up land in the study area indicates that it will continue in the future, and this will impact on the vulnerability of national food security. The government has anticipated this in some ways, including developing a food estate program outside Java Island. But, unfortunately, this effort has not yet shown satisfactory results. Thus, increasing the capacity and quality of planning, monitoring, and legal enforcement of spatial planning activities to achieve sustainable development is necessary. In an effort to control rapid LULC change from agriculture to urban areas, GEE and IA can be used to address data scarcity both rapidly and cost effectively. Further works needed is the use of GEE and IA to monitor LULC change for shorter time intervals as well for predicting future LULC change. These research results are expected to contribute to the activities of regional planners and can be applied in other regencies/municipalities in Indonesia and in other similar countries.

## Figures and Tables

**Table 1:** Administrative regions in the north coast region of West Java Province.

No.	Name	Area	
		km^2^	%
1.	Bekasi Regency	1,272.7	14.9
2.	Karawang Regency	1,920.4	22.4
3.	Subang Regency	2,176.3	25.4
4.	Indramayu Regency	2,086.6	24.4
5.	Cirebon Regency and Municipality	1,109.2	15.0
	Total	8,565.2	100.0

**Table 2:** LULC area for 2003, 2013, and 2020 for each category across the study area.

No.	Category	Area					
		2003		2013		2020	
		ha	%	ha	%	ha	%
1	Agriculture	764,574.22	89.31	756,698.39	88.39	743,727.65	86.88
2	Forest	8,974.93	1.05	6,632.29	0.77	6,328.22	0.74
3	Built-up	69,642.02	8.14	80,469.19	9.40	94,655.71	11.06
4	Shrub/bush	4,063.95	0.47	3,248.31	0.38	2,297.19	0.27
5	Water bodies	8,812.07	1.03	9,019.01	1.05	9,058.41	1.06
	Total	856,067.19	100.00	856,067.19	100.00	856,067.19	100.00

**Table 3:** Transition matrices for the two time intervals of the study, indicating persistent (in bold) and changed areas in hectare: (a) 2003–2013 and (b) 2013–2020.

	Category	Agriculture	Forest	Built-up	Shrub/bush	Water bodies	Total	Gross loss

(a)	2013 (ha)							
2003	Agriculture	**732,339.00**	1,068.38	27,439.80	2,548.14	1,178.90	764,574.22	32,235.22
	Forest	3,465.76	**5,370.15**	4.05	120.84	14.13	8,974.93	3,604.78
	Built-up	16,624.67	0.42	**52,989.26**	9.81	17.86	69,642.02	16,652.76
	Shrub/bush	3,294.88	191.73	7.74	**569.51**	0.09	4,063.95	3,494.44
	Water bodies	974.07	1.62	28.34	0.00	**7,808.03**	8,812.07	1,004.03
	Total	756,698.39	6,632.29	80,469.19	3,248.31	9,019.01	**856,067.19**	
	Gross gain	24,359.38	1,262.14	27,479.93	2,678.80	1,210.97		

(b)	2020 (ha)							
2013	Agriculture	**722,224.53**	1,579.56	29,927.63	1,863.01	1,103.66	756,698.39	34,473.85
	Forest	1,831.47	**4,702.68**	0.63	93.73	3.78	6,632.29	1,929.62
	Built-up	15,729.77	0.27	**64,711.53**	5.40	22.23	80,469.19	15,757.66
	Shrub/bush	2,866.10	40.67	6.03	**335.05**	0.45	3,248.31	2,913.26
	Water bodies	1,075.78	5.04	9.90	0.00	**7,928.29**	9,019.01	1,090.72
	Total	743,727.65	6,328.22	94,655.71	2,297.19	9,058.41	**856,067.19**	
	Gross gain	21,503.12	1,625.54	29,944.19	1,962.14	1,130.11		

## Data Availability

The data used to support the findings of this study are available on request.
